# Co‐production to understand online help‐seeking for young people experiencing emotional abuse and neglect: Building capabilities, adapting research methodology and evaluating involvement and impact

**DOI:** 10.1111/hex.13622

**Published:** 2022-10-10

**Authors:** Vanessa Bennett, Chloe Gill, Pam Miller, Asher Wood, Cassia Bennett, NeurOX YPAG, Ilina Singh

**Affiliations:** ^1^ Neuroscience, Ethics and Society Group, Department of Psychiatry University of Oxford Oxford UK; ^2^ NSPCC, Research and Evidence Team London UK; ^3^ Present address: Co‐production Collective, UCL Engagement, Bidborough House 38‐50 Bidborough Street, WC1H 9BT London

**Keywords:** children and young people, co‐production, emotional abuse, help seeking, involvement, neglect

## Abstract

**Background:**

Involving young people (YP) as co‐researchers (YCoR) in mental health research is important for ethical and epistemological reasons. However, approaches to involve and evaluate ‘meaningful involvement’ in complex qualitative mental health research, and how to evaluate impacts (or change) for the co‐researcher and the research is less well defined.

**Objectives:**

This co‐produced research explored the experiences of YP seeking help for emotional abuse and neglect via an online, peer‐peer message board. This practical case study aims to evidence the meaningful role and impacts associated with YCoR involvement in sensitive and complex mental health research using a flexible approach to co‐production.

**Methods:**

During the Covid‐19 pandemic, we explored on‐ and off‐line approaches and adapted research methodology to build relationships, knowledge, skills, and confidence with YCoR. The virtual involvement was evaluated against the five principles of co‐production. Anonymous, continuous digital feedback, reflective practices and multiple dissemination outputs are used to evaluate the impact of the study on those involved and the research.

**Results:**

Ten members of NeurOX Young People's Advisory Group were involved in the core project. Additional members were invited at later stages and in the dissemination of outputs. We describe a supportive, scaffolded learning approach to build capabilities and embed the lived experience of YCoR in complex qualitative research. A digital blended approach was acceptable to YCoR, principles of co‐production were met and the impact/benefits of involvement are described. To demonstrate the epistemological value of involving YP we evidence YPs capabilities for involvement and the ‘change’ or contribution YCoR made to the research through reflective practices.

**Conclusions:**

This case study demonstrates how flexible approaches co‐production with YCoR can be robust and responsive to balance ethical and epistemological impact in complex mental health research. Supportive, scaffolded practices and safe environments helped build the confidence and capacity of YCoRs to demonstrate valuable phenomenological insights in the analysis. YP's perspectives on how they describe ‘meaningful’ and impactful involvement illustrate the reciprocal benefits gained through working together.

**Public Contribution:**

This case study describes the YCoR involvement throughout the research and dissemination of outputs. YCoR co‐authors were involved in developing the outline and reviewing the draft stages of the manuscript.

## 1 BACKGROUND

Ethical and epistemological reasons for involving children and young people (YP) in mental health research have been widely reported.^1^ Ethically and morally, children and YP have the right to be involved in the research that affects them.^2^ Epistemological reasons require researchers to demonstrate how involvement benefits the quality and relevance of the research.^3^, ^4^, ^5^ Some researchers propose consideration be made to both ethical and epistemological reasons for involvement in research, but there is a tendency in evaluating public and patient involvement to focus on the generation of research knowledge and the positionality of the researcher, risking tokenism.^6^ This can be due to a host of challenges, including short‐term funding and a lack of clarity on what is required to demonstrate impact versus what is meaningful.^6^, ^7^, ^8^, ^9^, ^10^ What constitutes ‘meaningful involvement’ for YP is also still debated and the evidence of the impact for individuals seldom considered.

While acknowledging the rights of *all* children and YP to have a voice, and representing them in the research, this paper about the involvement methodology refers predominantly to YP as the young co‐researchers (YCoR; aged 14–18 years) since it is their perspectives embedded in this research. A pragmatic approach ‘to evaluate meaningful involvement’ could simultaneously respond to both tenets to enable and empower YP to develop personally, actively shape their environment and guide the research to incorporate experiences of the real beneficiaries of the research. In turn, this aims to improve research relevance.^5^, ^11^ Thus, valuing and demonstrating ‘meaningful involvement’ with YP may be better framed across three areas of impact: (i) striving to ensure the ethical rights of YP to be inclusively involved in research; (ii) the direct benefits for the YP involved in the research; (iii) the broader benefits of the knowledge generated for research, and how it may assert change for more YP, services or communities.^4^, ^9^, ^10^

Involving YCoR through flexible co‐production could resolve some of these tensions.^12^, ^13^ Co‐production as outlined by the NIHR involves five core principles: sharing of power; building and maintaining relationships; including all perspectives and skills; respecting and valuing each others' knowledge and reciprocity.^14^ The NIHR has more recently added further UK standards for public involvement: inclusive opportunities, working together, support and learning, communications, impact and governance to guide and support self‐reflection.^15^ Similarly, United Nations International Children's Emergency Fund (UNICEF) has reported standards for engagement and participatory involvement of children in low‐, middle‐ and high‐income countries which are helpful in assessing the quality of community engagement (and involvement) but are broad and could benefit from relevant practical exemplars.^16^ Laying out principles of co‐production and ethical standards is integral to good engagement and involvement with YP.^17^ However, exactly how to enact these through participatory and involvement methodologies, how to ‘judge’ whether they have been met, and how this relates to YCoRs interpretations of what is meaningful, is often not formally set out for research programmes.^18^, ^19^, ^20^

Direct benefits to YCoR involved as advisors and/or co‐researchers are rarely explored and described, and guidance on how to evaluate such involvement is less well developed.^18^ However, a recent rapid evidence review conducted with YP synthesized evidence and reported that ‘good involvement’ can enhance YCoRs sense of agency and empowerment to extend their learning and awareness about their own health; increase health‐related literacy for themselves and other YP; expand their skills and capabilities in other areas of life; increase career prospects (including an increased motivation to pursue health‐related opportunities); and, produce further benefits to broader communities of YP.^5^ Reported benefits to mental health research of YCoRs involvement include: ensuring the focus of studies starts and remains relevant to their experiences (and their peers) and priorities; better engagement and recruitment; meeting ethical standards^8^ and methodology (including data collection and analysis) that is more acceptable and responsive to YPs rights, needs and perspectives.^3^, ^7^, ^21^ Currently frameworks available, developed with public contributors, are the GRIPP2 reporting checklist and the Public Involvement Impact Assessment Framework.^22^, ^23^ These are helpful in knowing what to report and instilling thought processes but hold less utility for understanding how to implement or evaluate practices in accessible ways for co‐production with YCoR. Additionally, prescriptive guidelines and checklists may inhibit flexibility and creativity in levels of involvement, roles and methodological approaches to working with diverse YCoR and different communities that can be at the heart of effective youth involvement.^13^, ^19^, ^20^ Therefore, documenting procedures and evidencing what YCoR find meaningful to them in context—what they ‘get out’ of being involved in a research project—is important to evolve practices, evaluation and standards, and balance power.^12^, ^20^, ^24^ Case studies detailing different modes to capture the broader value, such as the difference that deeper incorporation of YCoRs phenomenological interpretations make to the research, may also motivate and overcome some structural and perceived barriers for researchers.^25^

Many studies have shown that YCoR can make valuable contributions in complex areas of health research but require the appropriate support, training and environment to do so.^18^ To be enabled and empowered, YCoR need to have sufficient knowledge and capacity to be meaningfully involved in qualitative research.^13^ Researchers must also be flexible as they develop methods that are responsive to capabilities and needs and meet the principles of co‐production.^13^, ^20^ Involving YP in the ‘how to’ by working with them to adapt research methodology that fully embraces their perspectives is critical.^3^ However, such practical approaches and implementation of qualitative methodology, and its evaluation, have sparsely been reported in mental health research and further case studies are needed.^9^, ^20^

This paper presents such a case study researching a complex area of help‐seeking through co‐production with an established Young Person's Advisory Group (YPAG)—the NeurOX YPAG. A YPAG is one model of involving YP in health research. Such models vary considerably; by levels of involvement, power sharing and decision‐making of YP throughout different stages of the research process.^24^, ^26^ The guiding principles for the NeurOX YPAG are reported in Pavarini et al.^3^ The involvement model applies the principles of co‐production and is flexible, with emphasis on transparency in decision making, to facilitate different roles and develop capabilities of YP across advisory work (single interaction), consultations (multiple interactions) and co‐produced research in mental health and ethics research.^3^, ^13^, ^21^

This co‐produced research aimed to explore the psychological characteristics and help‐seeking journeys of children and YP who have experienced emotional abuse and neglect (including experiencing/witnessing domestic abuse) and access the Childline message board service.^27^ This online moderated peer‐peer service facilitates any child or YP (aged 11–19 years) to connect with others and seek support for any safety, social or psychological worries. The Childline message boards are designed for children and YP, so ethically and morally, research on them should include them. With regard to the epistemological rationale, involving YCoR who have been/are potential service users seeks to incorporate their social, psychological, developmental and environmental experiences into the design of research and analysis, and recommendations for service development. This is particularly relevant and important to capture in this context through the methodological approaches (to involvement and research) as YCoRs perspectives on emotional abuse and neglect are largely absent from this specific area of research. YPs perspectives on emotional abuse and neglect have also been reported to be discordant with those of adults.^28^, ^29^, ^30^, ^31^, ^32^, ^33^, ^34^ Thus, involving YCoRs to analyse help seeker messages offers a more nuanced and contextualized interpretation and understanding of their peers' language and how children and YP frame emotional abuse and neglect, and enables inference of psychological states, barriers and facilitators to help‐seeking in these anonymous peer‐peer forums. Such analyses may offer valid insights across research disciplines; for example, clinical and forensic psychology, and social care research.

The trauma‐informed, co‐produced involvement approach taken intended to strike a balance to respect YPs rights, support YCoR needs and develop their knowledge in sensitive research. Offering autonomy while nurturing growth in the specific capabilities (relating to conducting the research) is suggested to make a positive difference to the relevance and impacts of the research. This case study aims to share practical insights and evidence the meaningful role that YCoR had through: (i) flexible involvement methodology to build capabilities; (ii) adapting research methodology to robustly embed lived experience and (iii) incorporating monitoring and evaluation to document the range of impacts these offer for individuals and the research.

## 2 METHODS

**Figure 1:**
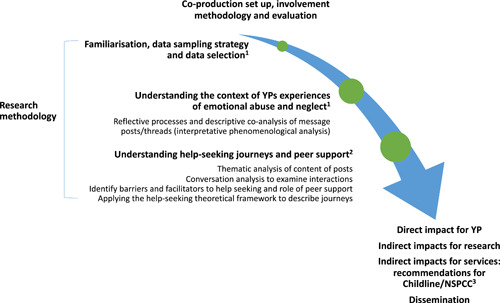
Overview of study elements. 1. Bennett et al.^35^; 2. Bennett et al.^36^; 3. Bennett et al.^27^

Figure 1 provides an overview of the different research methodologies that were adapted and applied for co‐production.

### 2.1 Funding, ethics and study design

The study was funded by the UKRI Emerging Minds UK network as a 3‐month cross‐sector research placement with the National Society Prevention of Cruelty to Children (NSPCC), and additional funding for involvement from the Department of Psychiatry, University of Oxford. The NSPCC provided additional support and funding for YPs involvement for dissemination. The study was approved by the NSPCC Research Ethics Committee (R‐20‐189, 2020) with reciprocal approval by the University of Oxford Central University Research Ethics Committee. All procedures complied with the approach in the ethics application to facilitate co‐production.

The ‘data’ analysed in the study were a series of text‐based messages posted on the Childline message board forming an online conversation (message thread) between anonymous (pseudonymised) help‐seekers and peer supporters. This ‘real‐world data’ is publicly available and anonymity and confidentiality were maintained in conducting this study.^37^ Service users are made aware their ‘data’ may be used for research and evaluation purposes to make service improvements while respecting privacy and avoiding harm. Involving YCoR offers an approach to assess and govern these sensitivities throughout the research and dissemination aspects. YCoR consented to the use of their data in dissemination activities. The short form and elements of the long‐form, GRIPP2 checklists were used to inform methodological approaches and reporting.^22^

### 2.2 Co‐production and scaffolded involvement methodology to build capabilities

**Figure 2:**
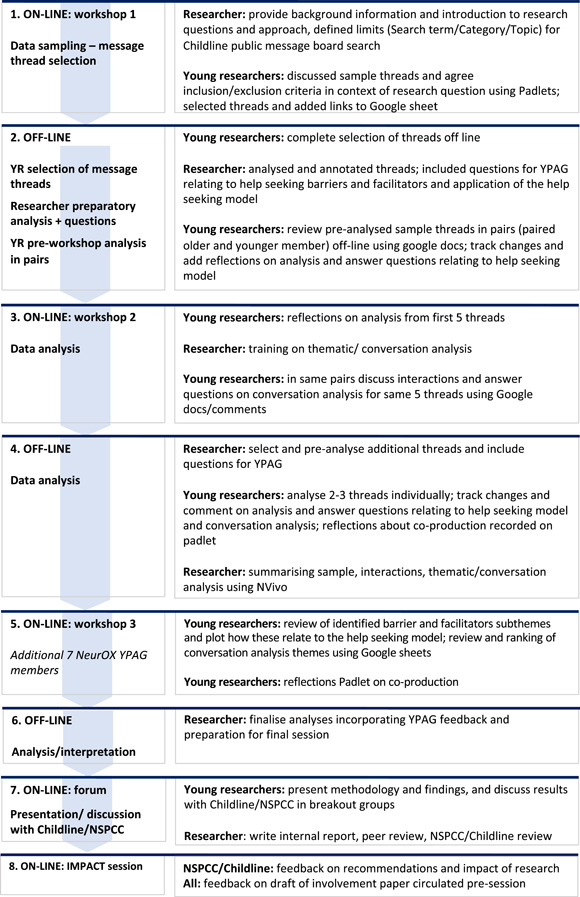
Overview of co‐production study design, roles and methodology

The study followed the principles of co‐production previously outlined in Pavarini et al.^3^ An outline of the study design, roles and involvement methodology is provided in Figure 2.

#### 2.2.1 Co‐production set up, recruitment from the NeurOX YPAG and reimbursement

Members of the NeurOX Young People's Advisory Group (YPAG), aged 14–18 years, were invited to apply to the Childline Message Board project. NeurOX YPAG members (and their parents) have previously consented to be involved and agreed to the terms of reference developed by agreement with the NEUROSEC Group, Department of Psychiatry, University of Oxford. The YPAG application process provided information about: the nature of the content posted on the Childline message boards (and web links), level of involvement, type of research and skills to be learnt, tentative dates, times/durations of meetings, anticipated pre‐ and postmeeting work and reimbursement. Signposting to mental health support was included due to the nature of the project. Eight YPAG members expressed an interest by the closing date (all were included) using a simple form. (Timelines to apply were initially short so four applied following a second recruitment call.) Two more YCoR were invited by the adult researchers to fill the 10 project places. All members of the NeurOX YPAG were later invited to join the third workshop; 15 YCoR joined this session. Thirteen members joined the final presentation and discussion with Childline/NSPCC, and nine members joined the dissemination session.

The 10 young project co‐researchers (YCoR) were White British, 2 of 10 YCoRs were male, and the rest were female or gender nonconforming. The majority attending state‐funded schools in Oxfordshire. YCoR included those with a range of lived experience of mental health challenges and a few had previously used the message board. One YCoR had been with the group for 4 years, remaining members had been with the group for almost 1 year. Half the group had previously been involved in some co‐produced research projects; five members had only been involved in an advisory capacity before this project. One adult researcher (V. B.) had recruited and worked with members of the established NeurOX YPAG, 9 months before study start on a range of advisory and co‐production projects.^21^, ^38^ The researcher had experience of recruiting and working with children and YP as research participants, and youth sector voluntary work.

It was estimated that the YCoR collectively contributed over 130 h to the research project over 3 months, and additional hours for dissemination activities (including during a paid work experience week). During the project, YCoR was reimbursed at a rate of £10/h of involvement, and those facilitating online sessions and later dissemination activities received £15/h.

#### 2.2.2 Focussing of research topic and safeguarding process

The initial framing for the project was to broadly explore the role of the on‐line peer‐support service in the help‐seeking journeys of YP. While naturalistic data offer many benefits, this presents practical and ethical challenges for designing research and robustly sampling data. Therefore, we chose to clearly define a help‐seeker population that could be stratified using the search function. This was informed by (i) the length of the research project (i.e., relative area of the board that could feasibly be sampled); (ii) evidence from the research literature (need and potential role that anonymous, confidential services play); (iii) choice of YCoR; (iv) the remits of the NSPCC/Childline service. Two areas that fulfilled (i) and (ii) were discussed with YCoR—self‐harm and suicide, or emotional abuse and neglect. In 1:1 telephone calls with an adult researcher (from NeurOX YPAG) following recruitment, these (or other) possible options for the focus of the research and any safeguarding concerns or worries relating to discussion of any sensitive content were discussed. YCoR were also encouraged to email any concerns or topics they would not feel comfortable reading, writing or talking about. The YCoR all expressed a strong desire to explore emotional abuse and neglect; they reflected on their perceptions of the NSPCC and Childline and considered the unmet needs of this population.

#### 2.2.3 Roles of YCoR and researchers, and levels of involvement

A detailed outline of the stages and levels of involvement is illustrated in Figure 2. The research team (YCoR, adult lead researcher and two NSPCC researchers) participated in three workshops and off‐line research, and two final sessions to discuss findings and recommendations, and dissemination of the research. YCoR were involved in analyses and provided reflection and interpretation at all stages, though did not perform the actual coding of data. This short study was conducted during school term time and the Covid‐19 lockdown, and involved complex coding using licensed NVivo software. Coding was therefore performed by one of the adult researchers (V. B.), and additional iterative strategies were employed to include YPs perspectives and allow reflexivity in coding to agree on reliable themes with YP (see Section 2.3.3).

Balancing power has been discussed with the NeurOX YPAG for previous projects and a hybrid online/offline model developed. Management focussed on: building a safe, respectful environment and relationships with adult researchers and each other; avoiding YCoRs being outnumbered by adult researchers (individual, paired and group working); use of online breakout rooms and Padlets facilitated by YCoR; and, feedback mechanisms. Minimal one:one work was carried out between the researcher and YCoR; unless requested by YCoR. Additional co‐facilitator responsibilities were agreed upon with two YCoRs, who led discussions in breakout groups. One female YCoR (member from 2016) had an experience with co‐production projects and co‐facilitating in the YPAG. Another male member had expressed an interest. A briefing call was arranged before the first session to prepare for the role. One YCoR was absent from the first Workshop due to illness but wished to continue in the project and was subsequently provided with the outputs from the first Workshop. They were briefed separately by the lead researcher via email exchanges. As agreed, another YCoR selected posts on their behalf from the sampling strategy. They joined all remaining activities and sessions.

#### 2.2.4 Blended virtual co‐production process and creating a safe, respectful environment

The research was conducted during the Covid‐19 pandemic (October to December 2020); therefore, a blended—virtual and offline—approach was developed (Figure 2). YCoR worked independently, in groups and in pairs over the 3‐month period. All Workshops were delivered using Zoom with breakout rooms. Other communications included telephone, email and a WhatsApp group. A range of online applications was used to facilitate the research including Padlets (available at https://en-gb.padlet.com) to facilitate discussions and capture feedback, google docs for the descriptive phenomenological co‐analysis, and google sheets for data sampling and later review of thematic codes from analysis and data interpretation.

Safeguarding arrangements and guidance for Zoom meetings previously developed with the NeurOX YPAG were provided to the YCoR. In the premeeting briefings and the first session, members of the research team were reminded of ‘house rules’ and Zoom features to ensure everyone felt able to contribute and encourage open sharing and respect for knowledge and experiences of all. During virtual workshops, the adult researchers were split across breakouts (for safeguarding). They remained with the camera off and provided minimal participation in discussions unless requested by the YCoR, or to provide practical facilitation and timing guidance (using Zoom chat).

#### 2.2.5 Building knowledge, capabilities and understanding throughout the research

Given the short duration of the study, the blended approach aimed to provide sufficient knowledge and develop skills through scaffolded learning. The information provided to YCoR is outlined in Table 1. Background material and presentations were provided before Workshop 1 and throughout for self‐directed research (including some scientific publications). Specific step‐by‐step training on qualitative research methods was provided. The aim was to satisfy different levels of motivation, cognitive abilities and ages, and facilitate YCoR's capabilities to be meaningfully involved and grow in confidence and research autonomy.^13^ Different tasks and various formats were trialled during the project to accommodate this and the digital approach; with continuous feedback and reflections from the YCoR guiding further sessions. Younger and older members were paired where possible.

**Table 1:** Information provided and approaches at several stages to scaffold knowledge and capabilities of young co‐researchers for research activities

Pre‐workshop	Project briefing and background information before first session included:
	Schedule of on‐line sessions, expectations, safeguarding guidance and details of reimbursement Summary briefing document—familiarization with Childline message boards, safeguarding information and overview of sessions Background reading suggested—NSPCC website sections on abuse Advanced copy of session PowerPoint slides
Workshop 1	Background information explained during workshop, include:
	Research population, rationale for the research and proposed research questions Definitions of abuse and neglect—from NSPCC website and extracted from research literature Potential gaps in research (and policy) relating to reporting of abuse of young people versus adults Theoretical help‐seeking framework (Rickwood et al.^39^) Barriers and facilitators to help seeking for adolescents for mental health problems reported by other researchers (Radez et al.^40^)
	Discussion and opportunity to raise questions
	Breakout session to discuss sample threads and compile inclusion/exclusion criteria
	Explanation of search strategy, individual selection of first few threads and discussion of queries before completing individual selection of threads off‐line
	Briefing on transcript analysis and help‐seeking questions
Off‐line	Emailed information briefing for off‐line tasks provided with researcher predescriptive analysis including embedded questions for pairs to work through in five pairs for first five threads
Workshop 2	Advanced copy of session PowerPoint slides send to YCoR
	Training presentation introduced qualitative approaches to data analysis focussed on the application of thematic and conversation analyses to the research
	Breakouts in pairs—YCoR applied conversation analysis to message threads they had previously reviewed and commented on
	Observations were discussed to ensure all understood the off‐line tasks
	Padlet for anonymous feedback on co‐production introduced
Off‐line	Each young co‐researcher individually received and analysed threads from their selection
	Padlet for anonymous feedback—continuous reflection
Workshop 3	Preliminary findings from thematic analysis were explained and the draft themes described
	In breakout sessions using google sheets, YCoR discussed their reflections on barrier and facilitator themes and their interpretations in relation to the help‐seeking model and role in interactions. Reflections were captured to amend themes
	YCoR reflected on how barriers and facilitators related to different phases of the help‐seeking process
	YCoR ranked the importance of conversational elements and qualities to help seeking and feeling supported
	The final presentation/discussion session with Childline members was planned by the YCoR and presenters assigned
Off‐line	YCoR co‐prepared a presentation off‐line with support from the lead researcher as necessary
	Padlet for anonymous feedback—continuous reflection

Abbreviation: YCoR, young co‐researchers.

To develop an understanding and provide a framework for the research methodology, other adolescent mental health help‐seeking literature describing barriers and facilitators^40^, ^41^ and a conceptual model of adolescent help‐seeking^39^ was explained to YCoR in Workshop 1. Questions related to this framework were incorporated into the methodological approach for the thematic analysis. Further questions were included to guide YCoR through conversation analysis at the end of each thread.

### 2.3 Adapting research methodology for co‐production

Research methodologies were adapted to enhance knowledge and skills and improve accessibility to facilitate the incorporation of YPs perspectives throughout the research process. Continuous YCoR informal consultation and opportunity for reflections, individually and as a group (online and offline), enabled researcher responsiveness and were integral to flexibility during co‐production as well as formal evaluation (see Section 2.4.2).

#### 2.3.1 Familiarization, approach to data sampling and message thread selection

In the first Workshop, YCoR were led through a series of activities by the young co‐facilitators in small breakouts (3–4 YCoR) to familiarize, review and discuss sample message threads. YCoR developed inclusion/exclusion criteria to help guide their selection. Padlets were used in breakouts to enable all to participate and record their thoughts. This and data sampling are reported in detail elsewhere.^35^, ^36^ All YCoR copied their selected message thread URLs into a Google sheet; these threads were then imported into NVivo (v12) by adult researchers.

#### 2.3.2 Role of YCoR and involvement process for the interpretative phenomenological co‐analysis

Message threads were pre‐analysed by the adult researcher (V. B.) before analysis by YCoRs. This was to provide a structure for the analysis pulling out: information about the type of abuse, contextual features, psychological characteristics, help‐seeking motivations, writing style, language and conversational features. In pairs, YCoR were emailed a brief and link to a Google doc with the pre‐analysed full thread and help‐seeking questions to respond to. YCoR commented and added their own reflections using tracked changes in Google docs before workshop 2. During Workshop 2, the group discussed analysis of the first thread to check understanding and address any questions. Following methodology training, YCoR pairs worked through a second set of questions on the same thread; this time relating to the interaction—conversation analysis. Following Workshop 2, YCoR analysed assigned threads individually.

#### 2.3.3 Involvement and incorporating YCoR perspectives in thematic and conversation analyses

YCoRs reflections from the co‐analysis and responses to help‐seeking questions were used by the adult researcher to guide reflective thematic analysis.^42^ A first‐generation thematic map was developed that identified barriers and facilitators to help‐seeking, and the potential role of peer support.

YCoRs reflections and responses to questions relating to conversational analysis explored the text‐based interactions between individuals over time; identifying features and qualities that may have a role in inhibiting or facilitating help‐seeking and feeling supported throughout the discourse.^43^, ^44^, ^45^, ^46^, ^47^ YCoR responses were then categorized and themed by the researcher.

During Workshop 3, YCoR discussed and commented on the identified themes for both analyses, and how they may relate to the different stages of the help‐seeking model. They also discussed the barriers and facilitators to help‐seeking relating to conversational elements and prioritized which of these were most important. Themes and a codebook were finalized by the adult researcher following this session. Full details of qualitative methodology and findings are provided in Bennett et al.^36^ For this short‐term project, reflective processes among the YCoR and with the researcher were used to guide coding and establish trustworthy themes and subthemes.

### 2.4 Finalizing recommendations, dissemination and evaluating impacts

#### 2.4.1 Feedback to NSPCC/Childline and role of YCoR in finalizing recommendations

During the final workshop (Workshop 3), YCoR agreed an approach to present and discuss findings with six senior and executive board members of Childline and NSPCC and three adult researchers. Two YCoR worked together off‐line, to build a slide presentation summarizing the research, and plans for breakouts to share and discuss results. Thirteen YCoR attended. YCoR led the breakout sessions with Childline and NSPCC staff and participated in a wider discussion in the main session. Recommendations were finalized incorporating these discussions.

#### 2.4.2 Evaluating principles of co‐production, blended involvement and impact for YCoR

To formally evaluate the blended on‐ and off‐line approach, how the research met the principles of co‐production and to capture what YCoR thought were the benefits to them, an anonymous Padlet was provided. This was introduced during the second workshop; YCoR were prompted to add reflections to open questions in/after each session. The questions are provided in Supporting Information: Appendix 1. As collaborative partners in the project, adult researchers (V. B., C. G., P. M. and J. J.) communicated informally (online and via email) on a regular basis to reflect on the project and support each other but no formal evaluation was incorporated.

#### 2.4.3 Involvement in developing outputs and dissemination activities

YCoR were invited to be involved in all communication outputs; involvement was a joint agreement commensurate with their skills and available time. Nine YCoR expressed an interest in being involved in further written outputs; six joined an additional virtual session in July 2021 to plan and develop outlines for an NSPCC Briefing Report and peer‐reviewed publications. YCoR provided further input during the paid Work Experience Week working with representatives from the NSPCC (Policy, Fundraising and Communications Teams) and off‐line via email correspondence. Four members worked offline with the adult researchers to co‐author publications over a 1‐year period.^35^, ^36^ Three YCoR participated in an interview with the NSPCC Press officer and approved a press release distributed by the NSPCC. One YCoR participated in an interview for BBC South today with one adult researcher (V. B.). Six members of the NeurOX YPAG supported the development of a research grant application based on the findings of this project (November 2021 to January 2022).

## 3 FINDINGS

Different approaches for reflection, feedback and evaluation of the YCoR involvement methodology were included in the study. Informal methods (online, offline, email and What's App—individually and in groups) throughout the project enabled responsiveness and facilitated the adaptation of the research to support YCoR involvement. The primary formal evaluation method, using Padlets (with an option for anonymity) captured more structured feedback following the second workshop; this evolved as the relationships and the ‘research environment’ was established.

### 3.1 Flexible involvement methodology to build capabilities

The blended co‐production approach to involvement during the Covid pandemic was evaluated. Using a Padlet, YCoR reflected on what had worked well, and not well, with the format and approaches to the research (Table 2). The blended process was welcomed by most YCoR in facilitating scaffolding exercises, peer‐peer learning, relationship building and shortening of on‐line sessions. Furthermore, the use of pre‐and postmeeting work supported the ability to learn, understand and build the capacity to engage with the research. Balancing the time commitment for this short, intense, project was the greatest challenge for all parties. For some, the blended approach, allowing time on‐ and off‐line to completed activities, appeared to alleviate this challenge. Scaffolding and learning opportunities appeared to provide sufficient support and understanding of tasks in the short time frame. From their reflections, these practices enabled YCoR to engage intellectually and emotionally throughout the research process. Repetitive feedback sessions from breakout groups were criticized and accordingly the process was adapted early on to avoid this.

**Table 2:** Feedback on blended on‐ and offline format and scaffolded approach to involvement

Pre‐ and post‐meeting work	‘The pre‐work was quite interesting which made it easier to do…’
	‘The pre and post work was good for the session. It allowed the session to flow better and for more things to be discussed. Also, the work before the meeting was very interesting’.
	‘I like the pre‐meeting work a lot as we're actively engaging in research and not just providing our opinions on the process’.
	‘I really like the pre/post‐meeting work—it was really interesting’.
	‘I liked how the post‐work was explained in the meetings and we started doing some together before going off and doing them independently—it ensured that everyone knew what they were’.
Use of breakout rooms	‘The breakout rooms were good too and focused questions made analysis run very smoothly’.
	‘Breakout rooms work great!’
	‘Especially the small breakout rooms were good’.
Overall format and flow of sessions	‘I think the meeting was balanced well there weren't really any deficiencies’.
	‘The meeting seemed to have good proportions’.
	‘The meeting flowed very well from start to finish’.
	‘Sometimes bringing back discussions to the group can feel tedious; especially if people are repeating the same points or agreeing with one another’.
Discussion session with Childline on implications of the research	‘I liked meeting the Childline people and learning about their work so it would be cool if we could similar things in future’.
	‘The meetings were structured well although I would have loved to have more time with the people from Childline in the breakout rooms I felt as though we could have covered so much more and I really enjoyed the conversations’.
	‘I loved that session. The most interesting thing was probably the questions they asked’.
	‘Because we could see which bits of the research they found most interesting, prioritized and wanted to talk more about’.
	‘It was a nice way to end the project as we summed up everything we had done and it made the project feel like an achievement’.
Balancing time commitments	‘Been a little difficult at times with school work but mostly fine and definitely worthwhile’.
	‘The time commitment is fine. Working before and after the sessions with a shorter session in the middle makes the time commitment seem less as it is more flexible’.
	‘The pre‐meeting work is manageable and can be done at any point before the meeting, so everything is very flexible!‘
	‘I think the time commitment was fine. It is good being able to do the pre‐ and post‐meeting work in our own time’.
	‘Sometimes the mid‐week meetings have been tricky to attend with school and revision’.
	‘Doing the meetings during a school week and also managing revision time for exams’.
	‘I think the length of the meetings is good and all the work has been manageable’.
	‘I enjoyed that the meeting wasn't too long and was still able to do lots of work’.
	‘It's been alright generally, just tricky on weekdays’.
	‘It's mostly been fine, sometimes a bit hard because of extra classes after classes’.

### 3.2 Adapting research methodology to build capacity and embed lived experience

The scaffolded co‐produced approach guided methodological development and aided the reflective approach. Reflective practices and reiterative processes between the YCoR and adult researchers using Padlets, tracked changes in Google docs and activities with Google sheets were integral to meaningfully capture the lived experience of YCoR. YCoR were able to add to, and challenge assumptions of, the adult researchers interpretations and respond to help‐seeking questions. The extracts in Table 3 demonstrate the capacity of YCoRs to understand the research and apply theories and methodology to incorporate their lived experience across the following themes.

1. *Analysing and interpreting complex text*. YCoR additions (underlined text) showed they were capable of analysing complex texts, extending and challenging the adult researcher's pre‐analysis to offer additional/alternative explanations relating to the context, potential lack of shared experience and validation. They also made suggestions about how to enhance knowledge around the seriousness of abuse to empower other YP to seek help.
2. *Applying theoretical frameworks*. YCoRs demonstrated their understanding of psychological characteristics, the impact of experiences and interpretation of the language used in response to embedded questions relating to the theoretical help‐seeking framework. Their responses showed a high level of understanding of psychological concepts and empathy for the help seeker's experiences; introducing psychological language and evidencing concepts such as ‘mental state’ and ‘dissociation’.
3. *Understanding and developing empathy*. Although some YCoRs had the experience of using the message boards, many acknowledged that the experiences of the population were different from their own. They noted that reading numerous message threads across the Childline Message Boards, during the initial familiarization exercise helped develop their understanding and empathy. The example illustrates the sympathy and the empathy YCoRs developed for the research population. They commented further on their development of empathy in their feedback (Box 1).
4. *Interpreting emotion and language*. YCoR offered valuable insights into language and the perceived impact of conversational approaches between YP. Extracts from different YCoR describe their interpretations of language; the compassionate qualities and their importance in the delivery of peer support and in building rapport and connection with others. Many help seekers used emoticons or ‘terms of endearment’ to end posts; YCoR suggested how a YP may interpret this in the context of the message board conversation.

**Table 3:** Examples illustrating capacity of YCoR and reflective methodology to embed lived experience in analysis of message threads

Example	Input of YCoR at different methodological stages*
1. Analysing and interpreting complex text	YCoR additions in tracked changes to a pre‐analysed message threads:
	The PS doesn't share any description or examples of their own experiences or own emotions relating to them. *This may be because while they empathise, they don't have enough information on the person to share relevant personal experiences*.
	Instead they demonstrate their interest and perhaps solidarity/empathy by referencing their post and the content of the post—mirroring their feelings back to them and telling them they understand/can imagine without judging.
	They validate that it does appear to be a form of abuse to the HS. *Again perceptively recognising the need to assure the person of their problem's seriousness*.
	*…the father is less conventional with his abuse which means that the help seeker is doubting whether they are really facing abuse, i'd imagine many people don't post at all because of this worry, perhaps it would be helpful for young people to be given resources that help them define what they're going through so they have the confidence to speak out about it*.
	*Young co‐researcher tracked changes denoted by italic text.
2. Applying theoretical frameworks: using the help‐seeking model	Responses of YCoR to embedded help‐seeking questions (for analysis of the first help‐seeker message)
	*Question: Where is the help‐seeker in the help‐seeking process?*
	‘I think they are at 4/3 on the help seeking model as, yes, they have the help but it may not have been effective as they are still in denial and unable to comprehend emotions. It has signs of being effective as they are able to comprehend the events that have happened to them’.
	‘A barrier may be the unwillingness to accept their mother has neglected them as it might relate to the fact she does not love them. Facilitators may be the fact that they do not live with their mum anymore so they do not have to face her anymore. She has realized that what their mum did has had serious mental repercussions on them/their sister’.
	*Question: What do you think may be most helpful to encourage help seeking/support for the help seeker, and why?*
	‘Asking questions not related to the statement they have already said would have been the easiest way to engage the help seeker. For example: what is life like now with your dad? How is your sister? Has your mental state improved since moving house? Also if they had a story of neglect themselves this might build a bond between the HS and the person who made the comment because they have something in common’.
	*Question: What do you think may be unhelpful, and why?*
	‘A story could trigger the help seeker of personal negative experiences which they choose not to mention in the post. Questions might be discouraging to the HS if, for example, their mental state has not improved or if they know their sister is not okay as a result of the neglect’.
	*Question: Can you suggest any potential barriers (what may be stopping them) and facilitators (what may be motivating them) to getting/continuing to get help?*
	‘Not wanting to remind themselves of the negative memories again as it could be upsetting—seems unlikely as the help seeker seemed to be able to dissociate from the experiences’.
3. Understanding and developing empathy	Responses of YCoR to embedded help‐seeking questions (following a peer supporter message who is replying to a help seeker)
	*Question: What do you think of the quality of their [peer supporter] reaction and how this may affect the interaction of this young person on the boards?*
	‘I think that there is quite a poor quality of reactions as they exhibit a lack of empathy and the small amount of information that they receive is not particularly useful to the help seeker’.
	*Question: Where do you think the help seeker is in the help seeking model/process? Are they likely to have progressed? Why/why not—what are the main factors?*
	‘I think the help seeker is at the first stage as they understand that they have a problem but are not able to articulate their emotions yet. I don't think that this interaction has helped them progress through the help seeking model as they still have not opened up about how they feel. This could be due to the cold, informative style of the responder’.
	‘…the help seeker is not able to use any of the suggestions and the responder has not helped the help seeker open up about their emotions’.
4. Interpreting emotion and language	Responses to questions in the conversation analysis about message threads
	‘The use of sympathetic and empathetic language is helpful because it immediately communicates that the responder is friendly and wants to suggest helpful advice. Near the end, they offer a concise message of encouragement to keep going, which makes it easy for the HS to remember and remind themselves of throughout the day. Signing off with the responder's name is a good attempt at establishing a personal connection’.
	‘Establishing a personal connection (by using names or anecdotes) is important for trust. If the HS trusts the responders, they are more likely to interact again and share more about how they feel. Colloquial language removes the background stress of structuring your point perfectly, so it encourages responses. Using quotes from the HS's post may make them feel listened to. Offering short yet detailed pieces of advice make the responses very clear. Adding questions for the HS at the end of the post’.
	‘It isn't used as an actual kiss. But more often used as a way to express that the messages tone is kind and soft or to show familiarity. In this context I believe that they are using it to show empathy to the HS. Or, it almost is an opening to a response (although it is not explicit) from the HS as the familiarity provokes a conversation’.

**YCoR describing the impact: Interview extracts between three YCoR and an NSPCC Press Officer about the project for dissemination activities (10 months after study completion)**

**Table:** 

Motivations	Why did you want to take part in the project?
Clara	I looked at the research and it looked like really interesting. Like a really interesting topic … that's basically the main reason I decided to kind of write why I would want to be a part of it.
Peter	I wanted to do it because it also looked like it would help. Childline is just a really big organization, so the fact that we were working with them meant it was going to be important and worthwhile.
Cassia	Because it was sort of improving a service which anyone could use. I hadn't really understood that it could be used for mental health. I thought it was more to do with abuse and stuff.

Research	What was the most memorable thing in the research process?
Clara	I think my favourite bit was when we went into detail about the threads and we looked at barriers and facilitators … like why people seek out help and [what] encourages them or discourages them to seek that help because there were some bits that I thought if I had just read this from this thread … then I wouldn't have picked up on these different aspects that could have helped or not helped the help seeker … and I thought it was really interesting 'cause you could see the effect once you kind of looked at it. You could see how this affected the help seeker and I thought that was one of the most interesting parts and it's one of the parts that stood out with need most. I thought the barriers and facilitators were really interesting and when we had to sort of pick apart like the things that they said and analyse them like that, I thought that was very interesting.
Peter	I thought it was pretty cool how we were able to kind of convert qualitative data into a spreadsheet of values. We did thematic analysis where we grouped … we came up with categories for common like threads through the messages. You tallied up in a spreadsheet how many times that [theme] came up … I never really thought about managing to convert kind of subjective things into more numerical data.
Cassia	I remember there was one thread where there were two … there was one help seeker and two helpers. A pair of supporters, I'm not sure. And you could see the difference in the styles that the help that the helpers were using. And it was things like … advice sometimes help, depending on how you phrased it. It was things like how quickly that people respond affects the help seeker and just all of these things. You could see it in the responses of the help seeker … and how that was affecting them and whether you could see the progress that the help seeker was making. And I found it really interesting because also the helpers could kind of reflect on what they had said, and then they would adapt their techniques, and that was just the progress and the adaptation that you could see was really, really interesting.

Involvement	Is there anything that stands out to you during that whole period of time that you were helping out with this research?
Clara	There were two things that are very different that I kind of thought of and one was just learning about ChildLine. It's like on the whole because in my head it had just been kind of oh 0800 1111. It makes sense that they have a website and they have all of these opportunities for people to seek help. I didn't realise quite how accessible that could be or how much it could help come and I didn't realise that there were all these resources, and to me that kind of helped me understand, so I would learn a little bit better and understand what it is [we are] actually trying to do and that it's not just a kind of phone helpline for people who are really, really struggling. But it can be so much more than that. And the other thing. This is more kind of in terms of research, but I think. I remember it being said that validation was one of the most important things to feel as a help seeker in the first stages of the help seeking model and that kind of … hit me because a lot of the people in the help seeking threads had kind of said what to me sounded like, really. They had described really horrible situations and then kind of said I don't know if this is normal or they were really questioning whether this was something to be upset about, and so it makes sense that validation was something that was quite important, but it really really hit me because it was quite emotive research.
Peter	It was interesting how the Childline people seemed to really take on board our suggestions and kind of ask us to critique … the message board service and come up with new things that it could do better.
Cassia	Well, I think something that stood out to me was uhm, in a good way. The amount of people who were willing to try and help other people and we're responding to other people's sort of comments in a really like mature and helpful way.

Lasting impact	Has the work that you did on the message boards inspired any of you in any way. You know almost a year on, and if so, how?
Clara	For me, I think maybe inspired is the wrong word, but it's got me thinking a lot more about kind of things that we've noticed in the research, especially about barriers and facilitators and it's got me kind of noticing. In kind of everyday life, what … could be detrimental to someone seeking help and just kind of little things, and I kind of come back to the research remembering. Kind of specific situations or not specific situations, but just remembering certain things that we noticed on the threads and it's just got me more aware. I think just in day‐to‐day life.
Peter	I didn't have anything particularly to add on top of that. I would just say. Agreeing with that, the main takeaway seems to be that there can be a lot going on, like behind the scenes. Which we wouldn't know about from just looking at somebody.
Cassia	Same as Clara, really the sort of issues that some people … that, I hadn't really known much about before. With like emotional abuse and neglect.

### 3.3 Evaluating multiple impacts of co‐production and dissemination

The reflective feedback Padlet captured many elements relating to co‐production and the benefits/impact for YCoR, as well as those aspects that did not work well. These findings are presented below and the questions asked with full feedback and further details are available in Supporting Information: Appendix 1. Questions around the Childline/NSPCC session were added for the end of the final session.

#### 3.3.1 Meeting the principles of co‐production

Evidence of the principles of co‐production from anonymous Padlet feedback is presented in Table 4. There appeared to be an adequate balance of power and decision‐making during the project. YP confirmed and qualified their perceived autonomy throughout the project. YCoR expressed that they felt comfortable with the ‘research’ environment, had developed trusting relationships within the team and the group was respectful of everyone's contributions. Demonstrating reciprocity is more complex to evaluate; requiring evidence of benefits to the YCoR, the researcher and the broader group. Some of this reciprocity is demonstrated throughout the feedback in Table 4 and also expanded below. Adapting the methodology may also have contributed to reciprocal benefits; supporting a reiterative methodological process. For example, notes from the familiarization exercises were used to guide the researcher's first analysis of threads which YCoR then commented on.

**Table 4:** Examples of how the project met the principles of co‐production

Principles	Examples of feedback on anonymous Padlet by young co‐researchers
Balance of power	‘Total autonomy because it's made clear that there's no stupid idea, question, point’.
Building and maintaining relationships	‘Lots of autonomy. Having the work written down helps as it means that tone is less likely to be conveyed swinging the points to either side’.
	‘Complete autonomy…’
	‘I like the pre‐meeting work a lot as we're actively engaging in research and not just providing our opinions on the process’.
Developing and including all perspectives and skills	‘I liked how the post‐work was explained in the meetings and we started doing some together before going off and doing them independently—it ensured that everyone knew what they were doing’.
	‘The pre and post work was good for the session. It allowed the session to flow better and for more things to be discussed. Also, the work before the meeting was very interesting’.
	‘Building on ideas for projects and reasoning my thoughts’.
Respecting and valuing knowledge of all those working together on the research	‘Everyone is respectful of all ideas’.
	‘Researchers are good at listening’.
	‘We were free to express any idea and it was a nice environment for discussion’.
	‘Learning from other people's thoughts and expressing my own’.
Reciprocity—benefits to everyone from working together	‘I think I have learned more about analyzing texts that are really emotional and deal with serious problems’.
	‘I have improved my perceptiveness when reading about others problems’.
	‘It was really interesting to read the peoples stories on Childline and I feel I have more awareness’.

*Note*: For all responses from the feedback Padlet, see Supporting Information: Appendix.

#### 3.3.2 Impact/benefits of the research for YCoR

YCoR reflected using the Padlets on the skills they have learned and reported many personal benefits. Members reflected on how they had developed personal skills and their confidence had increased, in that they felt more empowered to use their ‘voice’. The YCoR indicated that they had also extended their knowledge and awareness of mental health issues.

I think I have become more confident at speaking to people I don't know and voicing my opinions.

I feel like I have improved my discussion skills as well as becoming more aware of issues.

In addition, they felt more able to develop their analytical and interpretative skills to incorporate different perspectives and express themselves better.

Building on ideas for projects and reasoning my thoughts.

Learning about other people's thoughts and feelings and putting out your own thoughts.

They described how they appreciated their voices being heard by Childline/NSPCC representatives in the final session and how this made them feel. A sense of achievement and feeling they had ‘made a difference’ to others was important.

I enjoyed feeling that I had made a difference and helped improve the service.

The Childline/NSPCC session was great. It felt that as we were talking to people who were really closely tied to the process of improving the boards that what we were doing was really helpful and directly contributing. It was nice how they would listen to what we were saying and take it on board.

Adult researchers observed noticeable positive changes in the confidence of all participants with greater ‘virtual’ engagement and interaction and discussion as the project progressed. This was particularly evident in the final on‐line session with senior Childline and NSPCC representatives. With the full group (of 13 YCoR and 9 adults) many of the YCoR enthusiastically and confidently asked and responded to questions and actively shared their thoughts. In addition, the YCoR were proactive in sharing evidence and developing technical recommendations during this discussion.

Important to empowering YP is receiving feedback and the sense their voice can be translated to action. Recognising that the research is the first step in the process, the YCoR commented on what they would like in terms of the next steps and feedback.

It would be nice to hear about it if any of the changes we suggested are implemented.

It could be nice to see what/if they do with the research or their next steps for the information.

I would like to know if there was anything they disagreed with.

They also commented on further research questions or areas they wished to investigate as follows.

(1) The ongoing service user journey beyond the message boards (though they acknowledged challenges associated with this).
(2) Investigate the help‐seeking model further.
(3) Learn more by involving YP with experience of emotional abuse and neglect as YCoR.
(4) Involve more diverse YP, including broader age ranges, as YCoR.

In terms of the longer‐term impact of the project, three YCoR recalled specific details of their involvement and describe perceived benefits for them and others 10 months after the project finished in the NSPCC press briefing, Box 1.

### 3.4 Dissemination and broader impacts of the research and involvement

Following the final workshop, an internal report incorporating YCoRs recommendations was written, sent for peer review by the NSPCC and subsequently approved by Childline and NSPCC Executive Board. Six months after project completion (July 2021), representatives of the NSPCC (C. G. and P. M.) fed back to YPAG members. YCoR were invited to participate in further on‐ and off‐line work with the NSPCC's Research and Evidence and Fundraising Teams, and the regional Press Team to co‐produce outputs to disseminate the findings. The Department of Psychiatry (University of Oxford) also developed a news piece (leading to a BBC TV interview). In summary, communications about the study have included:

(1) Co‐produced NSPCC evidence briefing (4 YCoRs).^27^
(2) YCoR‐written presentation for High‐value Funders (10 YCoRs).^48^
(3) Press briefing with the NSPCC (see Box 1; 3 YCoRs).
(4) BBC South Today interview aired three times and mentioned on BBC Radio Oxford on 10 November 2021 (1 YCoRs, 1 researcher). The short clip delivered messages about the role of YP in the research and how they found the Childline Message Boards to be a ‘non‐judgemental space’ to support YPs mental health.
(5) Development of a collaborative, cross‐institution UKRI/MRC Grant Application to follow up on some of the research recommendations (6 YCoRs). Included two online sessions, a co‐produced two‐page young reviewer attachment and feedback from two NeurOX YPAG members on the full proposal. Submitted on 20 January 2022 (Council ref:MR/X003213/1), shortlisted (June 2022); funding declined (preparing alternative submission).
(6) Three peer‐reviewed publications (4 YCoRs).^35^, ^36^
(7) Storytelling (as part of a broader NeurOX YPAG evaluation).^49^
(8) Podcasts (youth‐led/produced as part of a youth‐led peer‐peer engagement programme).^50^

Perspectives and reflections of YCoRs and researchers on dissemination and involvement in publication development are included in Box 2.

**Perspectives on involvement in communication outputs and impacts**

**Table:** 

Eight of 10 YCoR from the initial project team were involved in communication outputs (some in more than one). All were sent the publication drafts for comment; all provided permission to include quotes and names. Four YCoR who wished to be involved in developing the manuscripts provided input at outline, two review stages, and responding to reviewer comments are listed as co‐authors for their respective papers. These are their perspectives on their involvement and perceived impact.
*Asher (co‐author, 18 years)*: As a young co‐researchers', my perspectives on being involved in communication outputs are centralised around ‘making a difference’. I think we are motivated by ensuring research is conducted in the most ethical and unbiased way; meaning, if we feel something could be made better by being done differently, constructive criticism will be raised! Input is most prominent during the designing stages of most projects (due to other commitments like education or family), though YCoR input does increase again during the analysis and review stages. Presenting findings closely related to their role in the research and including their perspective will clearly ‘make a difference’. Publication is an exciting stage for some of the group, though others aren't keen to be involved as much past the initial planning of communications. Young co‐researchers did not contribute as much to the actual academic writing and undertaken by the researchers themselves, though YCoR do understand the content of the papers thoroughly. They particularly enjoy working on tasks where they feel their input is valued and will directly benefit the outcome (such as co‐designing studies and gathering data); this is important to keep in mind when discussing how best to involve YCoR. Being placed in a group (video call) with other YCoRs (and not being outnumbered by researchers) aided in creating a safe and somewhat familiar environment in order for ideas to be discussed.
*Cassia (co‐author, 18 years):* I found that sharing our work was very rewarding and enjoyed the opportunity to talk about what we had achieved. I thought that the range of mediums and formats in which the research was shared was interesting and probably helped us reach a wider audience. It was rewarding to talk to other young people with a common objective to achieve something that would help other young people.
*Peter (co‐author of [40], 18 years):* My experience with co‐authoring manuscripts has been that my most valuable contribution can be in commenting on my own experiences, the collective young people's experiences, and the details of the study with which we were most closely involved. This is where I think young people can be most useful to communicating outputs, and it is with understanding the necessary lexicon, practices of, and generally what is expected of an output from a publication that the greatest challenge lies, as this is quite an unfamiliar territory. Though certainly it is very valuable to them and the output to involve young people in this academic side, this may be where they have to lean on researchers' experience more so than in other areas. Perhaps the best approach to involving young people is allowing them to contribute wherever as much as they feel they can and then correcting, reassuring and redirecting where necessary to help them learn the right way of doing things. This will help accommodate young people wanting or being comfortable with different levels of involvement.

## 4 DISCUSSION

While it is clear that YP has a right to be involved in research that affects them, it is less clear how to involve them and how to evaluate the impact on them and the research.^18^ This case study documents the involvement of YCoR in novel co‐produced research on online help‐seeking and peer support from the Childline message boards for YP experiencing emotional abuse and neglect.

This case study describes how flexible involvement facilitated good engagement enabling YCoR to build their understanding and capabilities to be involved in a meaningful way. An initial challenge was the need to implement a blended approach during periods of Covid‐19 lockdown. This was a disruptive and unsettling time for YCoR. However, the NeurOX YPAG had been involved in some virtual schooling and research before the study. Feedback from YCoR using a continuous anonymous Padlet to capture reflections, suggests that the environment and approaches worked well for most YCoR; offering flexibility to fit the research around school work. They found the structured scaffolding and supervised process informative, supportive and enabling throughout the analysis of this sensitive research. YCoR also noticed increases in their skills, abilities and understanding of research methodology and others' experiences, as well as enhanced knowledge about the role of peer support and Childline's role in supporting YPs mental health. A noticeable increase in their capabilities, confidence and ‘virtual’ engagement was evident to adult researchers as the project progressed. Flexibility, regular engagement, and online tools to facilitate the research were critical to motivate and building capacity. This approach aligns with the ‘rope ladder’ approach to participatory involvement and building capabilities proposed in Arunkumar et al.^13^

A second aim was to demonstrate how established qualitative research methods could be adapted and applied to accommodate the flexible online co‐production and ensure that YPs lived experience was embedded in the research outputs. This was achieved through a scaffolding step‐by‐step learning process. In keeping with true co‐production, research methods were developed and adapted with feedback around what worked through collaboration. A reflexive thematic analysis approach was chosen. This was achieved with: individual, paired and group reflective practices; group discussions; an exercise to refine and map the barriers and facilitators on the help‐seeking framework; and, reviewing and ranking themes identified in the conversation analysis.^36^ These reiterative processes allowed further incorporation of YCoRs perspectives to develop trustworthy themes for this project. These practices appear to have been important in capturing diverse phenomenological which may otherwise have been contradicted and lost to reliability analyses.^42^ Hence, this approach was justified and robust for this context. Other researchers have shown the value of adapting approaches to incorporate more diverse lived experiences through broader group participatory practices in interpretative phenomenological analysis.^51^ Some methodological limitations, and areas for further research related to data analysis were identified by both the adult researchers and the YCoR. These are discussed in Bennett et al.^35^, ^36^

Demonstrating ‘YPs capacity for involvement’ and the impact on research has been largely underexplored yet is an important factor in achieving meaningful involvement and developing rigorous research methodology. It is also very much the responsibility and skill of the researchers undertaking co‐production.^18^ The evidenced examples illustrate how YCoR, aged 14–18 years, were able to engage with this complex research throughout each stage of the research methodology using the adapted scaffolded approach. Tracked changes and embedded questioning in the co‐analysed threads demonstrated their confidence to originate, add to and, at times, challenge the assumptions of the adult/lead researcher. Through this process, they offered valuable insights relating to the psychosocial context of YP that could be missed or misinterpreted due to unintentional biases of an adult researcher. Their capability to notice the empathic qualities of the peer supporters and potential impact on help seekers was also evident in YCoRs research commentaries. However, YCoR highlighted the limitations of the research and their own difficulties in analysing the experiences of others that were quite different from their own, identifying their own biases as a potential limitation and making recommendations to include such perspectives in further research.

The final aim was to monitor and evaluate meaningful involvement and impact: for the YCoR involved, for the research knowledge generated and the broader implications of the research. Informal ongoing feedback via individual and group communication (online and offline) facilitated a flexible co‐production approach to meaningfully involve YCoR in complex research. Regarding the impact on YCoR and the research knowledge, reflective practices evolved throughout the study and enabled YCoR thoughts about the involvement process and their perceptions of their capacity for involvement to be documented on a continuous anonymous Padlet. This formalized yet ‘open’ question approach may have offered more space for reflection and collaborative exploration than surveys or checklists; including constructive and nuanced perspectives about what was meaningful and had changed for them to support research and trauma‐informed responsiveness. The approaches taken should be discussed and co‐developed with YCoR.

From the Padlet and Press Release reflections, YCoR invested themselves intellectually and emotionally in the research and reported how the principles of co‐production were met. YCoR also communicated what was most meaningful about being involved; they expressed individual benefits to their personal development and perspective taking as well as the knowledge and skills they had developed. Overall, they expressed satisfaction in being listened to, making a potential difference to service improvement, and ‘helping their peers’ who have had distressing experiences. This echoed through their willingness to be involved in further outputs, and the content of these.

Limitations and learning points to the evaluation of impact and communication outputs are proposed in Box 3. From the approaches used, feedback was generally positive. It is important to note that this short, structured study involved an established YPAG in a sensitive under‐researched area that they felt important^3^ during a period of Covid‐19 lockdown. YCoR commented that this project gave them some structure in their lives, as well as social contact and made them feel positive at a time when education was uncertain, schooling was limited, and they were socially isolated. Establishing a YPAG can also be a primary challenge requiring time to build rapport and relationships, as other researchers have reported.^20^ In contrast, this group had interacted on other projects before the study. These aspects may help explain the lack of challenge reported in evaluations. For longer projects, and different contexts, other challenges in terms of maintaining motivation, time and educational pressures need to be factored into planning, communication and evaluation. This is consistent with other researchers' experiences and guidance for working with young researchers,^5^, ^17^, ^20^ and NeurOX YPAG experience on other advisory and co‐production projects (also see YCoR perspectives on www.neuroxypag.org).^21^, ^51^

Box 3. **Key learnings and recommendations relating to feedback, evaluation and communications**

**Table:** 

*Informal evaluation methods*	*Learning points*
Started informally while developing methods and approaches alongside scaffolded learning. Feedback focussed at the start of the project on how to adapt the methodology, check and respond to capabilities. Included verbal feedback in sessions (and breakouts), email, What's App.	Evolved to monitor the involvement approach, research methods, the environment and communications. Allowed for early responsiveness without barriers associated with formality. Changes made early to respond to areas for improvement could have affected ‘overall evaluation’. Offered potential positive impact on experiences throughout the study; enabled continual progression and change. Flexibility was aligned with principles and approach to co‐production; allowed adaptations of research methods and checking of capabilities. Different options enabled some individuals to get 1:1 support (or in pairs) in relation to the ‘analyses’ and building capabilities.
*Recommendations*	
Create a sense of safety early on by normalising the sharing of views and feedback (with their peers and the adult researchers). Include different informal feedback methods throughout to facilitate support (and safeguarding), responsive monitoring, methodological development and building capabilities. Consider different approaches that facilitate negative feedback in ways comfortable for YCoR. Consult at the start of the project on communication approaches.	
*Formal evaluation methods*	*Learning points*
More formal methods evolved through digital sessions and research approaches. Anonymous Padlets with open semi‐structured questions. Open questions were constructed by the adult researcher (V.B); include an open ‘Anything else to add’ question which was not utilised by YCoR. Questions were added to Padlets following each session. Tracked changes to the research ‘content’; transparent process (using google docs and google sheets) so YCoR could see impact. Final workshop using spreadsheet exercises with YCoR to enable them to visualise and capture their changes to the research outputs.	‘Formal yet flexible’ evaluation aligns better with principles of co‐production; flexibility to adapt methodology and respond to different needs but provide some structure. Padlets allowed an anonymous, reflective space. Despite anonymity, YCoR may not have wished to report negatively; teasing out negative feedback requires further thought and consultation with YCoR. As posts are visible to all YCoR; there may be a confirmation bias with successive posts. However, the Padlet approach does allow for co‐reflection; responses had varied as well as common components, so there did appear to be a mix of confirmation, reflection and extension on other YCoR comments. (this may be dependent on the ‘safeness’ of the environment). Involvement of YCoR in constructing questions may be beneficial but may also lead to positivity bias (e.g., if they genuinely want the project to be successful). Tracking changes helped to document and show YCoR the difference they made to the research. Tracking changes provided an indicator of how effective the research methods are in incorporating lived experience and showed the ‘difference they made’, which is important to demonstrate impact for YCoR and the research. Group workshops using spreadsheets supported the iterative processes and illustrated how the YCoR perspectives were incorporated, enhanced autonomy and they could directly observe ‘changes’ they make.
*Recommendations*	
Discuss these approaches and limitations with YCoR at the start of the project. Openly discuss the ‘safeness of the environment’ and how to capture different elements of the feedback, including what will make a difference to them. Combine with informal, early evaluation.	
*Methods of engagement, feedback and evaluation*	*Learning points*
Offered different means of communication for YCoR to feedback about their experiences (individual and as a group). Used a continuous Padlet (instead of surveys). Anonymity was included as an option. Considered environment and peer support.	The environment and relationships (researchers and ‘peer support’) needed to feel safe and supportive for YCoR to feedback. Offering a mix of feedback mechanisms allowed flexibility for individual YCoR to decide how to feedback in a way that was comfortable for them. Collecting formal feedback can be challenging for the researcher so different approaches, reminders, and making time and space when together was important Potentially, better response rates and quality of responses can be achieved using digital approaches such as Padlets (compared with surveys on previous projects with same group). Some YCoR chose to be anonymous; others did not—accommodating preferences and change.These changed as YCoR became more comfortable. Feedback (and ongoing involvement) from the NSPCC on their recommendations was welcomed and very important for the YCoR; to feel they ‘made a difference’. Deciding together what will ‘make a difference’ up‐front should be a key focus of evaluation and may drive better engagement throughout based on YCoR feedback.
*Recommendations*	
Consult early, and throughout the project, on what methods will engage YC, what will help the project and what feels ‘safe’. Agree goals of evaluation up front; including ‘what will make a difference to them’. Co‐produce a safe supportive peer environment and a safeguarding protocol. Agree on the ‘Terms of engagement’ with YCoR (or co‐develop if not already done). Discuss how, when and who they would like to feedback the broader ‘impact’ of their research.	
*Involvement in communications (and relation to impact)*	*Learning points*
Communication opportunities evolved and expanded after the research component of the study was finished. A broad range of outputs were developed. Consulted YCoR as opportunities arose. YCoR discussed the project in other communications about their experiences of involvement (e.g., storytelling project and youth‐led podcasts).	This was a learning process for all and YCoR preferences evolved. Individual YCoR had different communication preferences; and some different to researchers (e.g., they were more motivated than the researcher to be involved in a TV interview). YCoR were more motivated towards communications when they understood ‘what difference’ it could make (relevant to YPs contexts). YCoR were able to understand the content of peer review publications with support and provided valuable comments but acknowledged some limitations: technical nature, time constraints, educational pressures, motivations. Some YCoR appreciated the benefits from being involved in research Grant development (6 YCoR) and publications (4 YCoR), as well as personal benefits. Note: this group of YCoR had worked together for 3 years across many projects (see podcasts). YCoR commented that peer‐reviewed publications and Twitter are more for the benefit of research/adult researcher beneficiaries (they were not motivated to prepare Tweets or Twitter threads about their involvement). Contributions towards further knowledge generation (i.e. more research or research papers) may be too far removed from what some YCoR perceive as meaningful or ‘making a difference’ for them to be motivated to be involved.
*Recommendations*	
Discuss communications and objectives at the start but also revisit as opportunities arise and the meaning of the project transpires. Be transparent about what the benefit is, and who/what this will make a difference to. Be clear on what is reasonable to expect regarding YCoR roles and rewards from involvement in co‐authoring peer‐review publications by agreeing this with them.

Regarding dissemination and impact beyond the study, finding a broad range of opportunities for YCoR to be involved in communicating the research may ensure youth voice is included and potentially enhance the benefits of their involvement and the outputs to public beneficiaries of the research (e.g., other YP and message board service users). Many opportunities were developed through the strong cross‐sector partnership, and at a low cost. Importantly, motivations and capabilities for types of outputs are different for YCoR and researchers. In terms of evaluating the impact of research and meaningful incorporation of youth voice beyond the individual and specific generation of research, knowledge is more difficult to assess. Ideally, researchers should aim to track feedback and changes to the research to demonstrate the different areas of impact—for YCoR, for their peers, for the research and broader implications that may lead to change (e.g., funding, policy recommendations).

## 5 CONCLUSIONS

Flexible involvement with supportive, scaffolded practices, investment in relationships and safe environments, and adapting research methodologies helped build the confidence and capacity of YCoR. This enabled YCoR insights to be embedded in the research and produce direct benefits for them. This paper provides a balanced ethical, epistemological and practical case study with different approaches to evaluating the impact of flexible, yet ‘robust’ co‐produced public mental health research with YCoR on sensitive topics. We propose that methodology can be adapted to be both reliable and responsive in incorporating the voices of those with lived experience and hence contribute to meaningful and impactful research.

## CONFLICT OF INTEREST

C. G. and P. M. are employed by the NSPCC. The remaining authors declare no conflict of interest.

## ETHICS STATEMENT

Reimbursement for YCoR was provided by the NSPCC. The study was approved by the NSPCC Research Ethics Committee (Ref:R‐20‐189, 2020) with reciprocal review by the Secretariat of the University of Oxford Medical Sciences Interdivisional Research Ethics Committee (Ref:R62044/RE001).

## Supporting information

Supporting information.

## Data Availability

The data that support the findings of this study are available from the corresponding author for journal peer review. Some data are not publicly available due to privacy or ethical restrictions.
