# Supplementary material for: Co‐production to understand online help‐seeking for young people experiencing emotional abuse and neglect: Building capabilities, adapting research methodology and evaluating involvement and impact
Source: Health Expect. 2022 Oct 10;25(6):3143–63. doi: 10.1111/hex.13622 (PMC9700148; doi:10.1111/hex.13622)
Supplement: Supplementary file 1 — Supporting information. [file HEX-25--s001.docx]

**Appendix. All anonymous responses to feedback questions recorded on reflective Padlet.**

| **Questions and responses on Padlet – available after Workshop 1** |
| --- |
| **What has worked well with the format of the meetings, pre-work and post-work for you?**  “The pre-work was quite interesting which made it easier to do. The breakout rooms were good too and focused questions made analysis run very smoothly.”  “The pre and post work was good for the session. It allowed the session to flow better and for more things to be discussed. Also, the work before the meeting was very interesting.”  “I like the pre-meeting work a lot as we're actively engaging in research and not just providing our  opinions on the process. Breakout rooms work great!”  “I really like the pre/post-meeting work – it was really interesting. Especially the small breakout  rooms were good.”  “I liked how the post-work was explained in the meetings and we started doing some together before going off and doing them independently - it ensured that everyone knew what they were doing.”  “I think the length of the meetings is good and all the work has been manageable.”  “I enjoyed that the meeting wasn't to long and was still ble to do lots of work.” |
| **What has not worked so well with format, structure, content of meetings, pre-work and post-work for you?**  “Everything has worked fine for me.”  “Sometimes bringing back discussions to the group can feel tedious; especially if people are repeating the same points or agreeing with one another.”  Sometimes the mid-week meetings have been tricky to attend with school and revision.”  “Doing the meetings doing school week and also managing revision time for exams.” |
| **How have you found managing the time commitment?**  “Been a little difficult at times with school work but mostly fine and definitely worthwhile.”  “The time commitment is fine. Working before and after the sessions with a shorter session in the middle makes the time commitment seem less as it is more flexible.”  “The pre-meeting work is manageable and can be done at any point before the meeting, so everything is very flexible!”  “I think the time commitment was fine. It is good being able to do the pre and post – meeting work in our own time.”  “It’s been alright generally, just tricky on weekdays.”  “It's mostly been fine, sometimes a bit hard because of extra classes after classes.” |
| **What have you found most challenging? did you feel this was manageable? was there anything that could have helped with this?**  “The most challenging was probably the finding the messages in the first place as there weren't many that fitted the criteria. Not sure what could've be done to help with this, it wasn't that big of a deal anyway. “Most manageable was brainstorming and presenting ideas to the group.”  “I think it was sometimes hard to analyse the message boards as the HS don't always give lots of details so it can be hard to understand what they are thinking.”  “It’s been fine.”  “It seemed completely fine and manageable. The most challenging thing would most likely be opening up with ideas.” |
| **What was your favourite session/or part of a session? What was most interesting about it?**  “I don't think I can pick a particular one but I loved the discussions.”  “It's always fun discussing but also learning about the final product from the project.” |
| **What would you have liked to do more of?**  “I think the meeting was balanced well there weren't really any deficiencies. I liked meeting the Childline people and learning about their work so it would be cool if we could similar things in future.”  “The meeting seemed to have good proportions.”  “The meeting flowed very well from start to finish.”  “The meetings were structured well although I would have loved to have more time with the people from Childline in the breakout rooms I felt as though we could have covered so much more and I really enjoyed the conversations.”  “Updates on the project.”  “It would have been nice hearing from other people’s thoughts (however they weren't able to go which is fine).” |
| **How much autonomy do you feel you have had to express your views and provide your thoughts on the research?**  “Total autonomy because it's made clear that there's no stupid idea, question, point. Researchers are good at listening.”  “Lots of autonomy. Having the work written down helps as it means that tone is less likely to be conveyed swinging the points to either side.”  “Complete autonomy. Everyone is respectful of all ideas.”  “Plenty of autonomy, we were free to express any idea and it was a nice environment for discussion.”  “I'm fairly confident with expressing my thoughts and ideas so it hasn’t been a problem.”  “I don't mind putting out my thoughts it's more just worrying about saying the wrong thing.” |
| **What other benefits do you feel you have gained from being involved?**  “Was nice to talk to people I don't see daily so that was enjoyable.”  “It was really interesting to read the peoples stories on Childline and I feel I have more awareness.”  “I enjoyed feeling that I had made a difference and helped improve the service.”  “I feel like I have improved my discussion skills as well as becoming more aware of issues.”  “Learning about other peoples thoughts and feeling more comfortable putting out your thoughts.” |
| **What knowledge and skills do you feel you have gained?**  “I learned from the Childline people about the way they moderate content. That was interesting and new to me.”  “I think I have learned more about analysing texts that are really emotional and deal with serious problems.”  “I have improved my perceptiveness when reading about other’s problems.”  “I think I have become more confident at speaking to people I don't know and voicing my opinions.”  “Building on ideas for projects and reasoning my thoughts.”  “Learning from other people’s thoughts and expressing my own.” |
| **Any other comments or suggestions?**  [No responses] |
| **What other feedback would you like from the researchers?**  “It would be nice to hear about it if any of the changes we suggested are implemented.”  “It could be nice to see what/if they do with the research or their next steps for the information.”  “I would like to know if there was anything they disagreed with.”  “I think there's a good level of feedback.”  “There isn’t much more feedback that I'd like.” |
| **Added to Padlet after final session with Childline/NSPCC** |
| **What would be your next research question following on from this project?  What would you most like to investigate further?**  “It would be interesting to see some of the analytics that one of the Childline people was talking about, where you can see the journey a user has made through using the message boards.” “Although I expect this not to be possible.”  “If I could, I would try to investigate how the message boards help the HS and investigate the HS model.”  “Probably the people who were in the project, find out more out how it felt or have a  different age gap.” |
| **How did you find the Childline/NSPCC session?**  “The Childline/NSPCC session was great. It felt that as we were talking to people who were really closely tied to the process of improving the boards that what we were doing was really helpful and directly contributing.”  “It was nice how they would listen to what we were saying and take it on board.”  “I loved that session. The most interesting thing was probably the questions they asked because we could see which bits of the research they found most interesting, prioritized and wanted to talk more.”  “It was a nice way to end the project as we summed up everything we had done and it made the project feel like an achievement.” |
| **What other feedback would you like from Childline/NSPCC?**  “It would be nice to hear about it if any of the changes we suggested are implemented.”  “It could be nice to see what/if they do with the research or their next steps for the information.”  “I would like to know if there was anything they disagreed with.” |
